# Indirect Genetic Effects for Growth in Pigs Affect Behaviour and Weight Around Weaning

**DOI:** 10.1007/s10519-018-9911-5

**Published:** 2018-06-19

**Authors:** Irene Camerlink, Winanda W. Ursinus, Andrea C. Bartels, Piter Bijma, J. Elizabeth Bolhuis

**Affiliations:** 1 Adaptation Physiology Group, Wageningen University and Research, Wageningen, The Netherlands; 2 Animal Breeding and Genomics, Wageningen University and Research, Wageningen, The Netherlands; 3 Animal Behaviour & Welfare, Wageningen Livestock Research, Wageningen University and Research, Wageningen, The Netherlands; 4 Institute of Animal Husbandry and Animale Welfare, University for Veterinary Medicine Vienna, Veterinärplatz 1, 1210 Vienna, Austria

**Keywords:** Pig, Weaning, Indirect genetic effects, Breeding, Genotype-by-environment, Enrichment

## Abstract

Selection for indirect genetic effects (IGE), i.e. the genetic effect of an individual on a trait of another individual, is a promising avenue to increase trait values in plant and animal breeding. Studies in livestock suggest that selection for IGE for growth (IGEg) might increase animals’ capacity to tolerate stress. We assessed the effect of a stressful phase (weaning) on the behaviour and performance of pigs (n = 480) divergently selected for high or low IGEg. High IGEg pigs were significantly slower to explore the feed and gained less weight than low IGEg pigs in the days after weaning. In line with previous findings, high IGEg animals may have prioritized the formation of social ranks.

## Introduction

Group living individuals inevitably affect each other through their social interactions. Social interactions are partly genetic, and this genetic component is known as an indirect genetic effect (IGE). IGEs, also termed associative effects or social genetic effects, are the genetic effects of an individual on another conspecific (Griffing 1967; Moore et al. 1997; Wolf et al. 1998; Muir 2005; Bijma et al. 2007). IGEs have become an established concept in behavioural ecology (Bailey et al. 2017), livestock breeding (Ellen et al. 2014) and plant breeding (Bijma 2014). IGE models capture the genetic effect of an individual on the performance of another individual in the same group (in case of animals) or vicinity (in case of trees). Thereby IGEs for performance traits contribute to the accuracy of the breeding value predictions and can increase the progress in the population’s performance without the need for additional phenotyping beyond the main trait of interest (Bijma et al. 2007; Bijma and Wade 2008). Hence, incorporating IGEs in the breeding strategies has attracted much interest in agriculture (Ellen et al. 2014).

IGEs do not relate to one specific social interaction, but instead capture the overall effect of several social interactions between individuals on a specific trait of the recipient individual. This can relate to any form of interaction between individual organisms, including social behaviour in animals (Ellen et al. 2014), competition for light, water and nutrients between plants (e.g. Muir 2005; Costa e Silva and Kerr 2013) or transmission of disease (animals: Lipschutz-Powell et al. 2012; forest tree: Potts et al. 2013).

Selection on IGEs is always for a certain trait, for example the IGE for survival (e.g. Peeters et al. 2012), pheromones (Duijvesteijn et al. 2012), or for growth performance (Bergsma et al. 2013). The IGE for growth, here abbreviated as IGEg, is the genetic effect of an individual on the growth performance of its group mates. In a one-generation selection experiment in which pigs where divergently selected for IGEg, animals selected for high IGEg showed behavioural changes related to a reduction in aggressive and non-aggressive biting behaviour (Camerlink et al. 2015). They appeared to be less fearful in behavioural tests (Reimert et al. 2014a) and their immune status suggested a lower stress susceptibility (Reimert et al. 2014b). Because of the range of behavioural and physiological changes related to the attempt to cope with stress, it has been suggested (in Camerlink 2014) that selection on IGEg might alter stress sensitivity. Stress sensitivity may, therefore, be an underlying mechanism explaining the difference in social behaviour between pigs with diverging IGEg. We here address the relationship between IGEg and stress sensitivity, which has to our knowledge not been studied previously.

In current pig husbandry, pigs face many stressful events such as handling by humans, weaning from the sow, relocation to an unknown environment and mixing with unfamiliar conspecifics (Martínez-Miró et al. 2016). Here, individual differences exist in stress sensitivity (e.g. Bolhuis et al. 2004; Ursinus et al. 2013). Pigs are particularly challenged when weaned under commercial husbandry conditions (Campbell et al. 2013). Weaning includes the separation of the young from the mother and in pig husbandry this often coincides with rough handling by stock workers, transport, a new environment, novel feed, and the first encounter with unfamiliar conspecifics. As a consequence, piglets often show a behavioural and physiological stress response in the first days after weaning (Oostindjer et al. 2011a). Typically, piglets show a reluctance to eat, a decrease in growth or even a loss in body weight and restlessness in the early post-weaning period. Housing conditions may alter the response to weaning, with in general a better adaptation to the new situation when enrichment material that encourages exploration, such as straw, is provided (Melotti et al. 2011; Oostindjer et al. 2011b, c). Exploration will aid in getting the animals familiarized with the new surroundings, including where to find feed and water, and can therefore reduce stress and increase feed intake (Oostindjer et al. 2011c).

We hypothesized that animals selected for positive IGEg (higher IGEg than population average)—i.e. estimated to have a positive influence on the growth of their group mates—would cope better with the stressful conditions surrounding weaning as compared to pigs with a negative IGEg (IGEg below population average)—i.e. estimated to have a negative effect on the growth of their group members. We expect an additive effect of the housing conditions, with a better coping response in animals housed in straw enriched conditions. Here we studied the ability of pigs to cope with the stressful conditions surrounding weaning, measured by their behaviour and weight, using 480 pigs that were divergently selected for IGEg and housed in either conventional barren pens or straw enriched pens after weaning.

## Methods

The experimental protocol was approved by the Institutional Animal Care and Use Committee of Wageningen University (Protocol Number: 2010055f), the Netherlands. The protocol was in accordance with recommendations of the European Guidelines for accommodation and care of animals.

### Animals and housing

The animals in this study were the same as in Camerlink et al. (2013) and Ursinus et al. (2014a). Eighty sows (Topigs-20) were serviced by Tempo boars to create a contrast (high vs. low) in estimated Indirect Genetic Effects for growth (IGEg). Detailed information on the genetic estimates can be found in Camerlink et al. (2013, 2014). In short, high IGEg offspring (40 litters) were expected to increase the growth of their pen mates, whereas low IGEg offspring (40 litters) were expected to decrease the growth of their pen mates. Dams and sires with the most extreme high and low IGEg of the available population were selected, and their offspring were studied. The contrast between high and low IGEg offspring was 14 g/day average daily gain (ADG) (based on six pigs per pen, (6 − 1) × 2.8 g/day = 14 g / day; Camerlink et al. 2015). Offspring (*n* = 1210) were born over five consecutive batches (i.e. farrowing groups).

Sows and piglets were kept at the experimental farm of TOPIGS Research Center IPG in Beilen, the Netherlands. A detailed description about housing and management can be found in Camerlink et al. (2013) and Ursinus et al. (2014a). Briefly, piglets were kept in barren conventional farrowing crates (3.8 m^2^ with 53% slatted flooring). If the number of piglets in a litter exceeded 14 then fostering was applied within the same treatment group. Males were castrated before 5 days of age for production purposes. Water was available *ad libitum* via a drinking nipple and after one week piglets had access to a commercial diet.

Piglets were weaned at four weeks of age. At weaning all piglets were weighed and assessed for health. A total of 480 piglets were selected for follow up based on absence of clear disease symptoms, sex (1:1 ratio), and backtest response (see below). Selected piglets (*n* = 480) received an ear tag with identification number and were then transported over a 2.5 h truck journey to experimental facilities “De Haar”, Wageningen, the Netherlands. During transport, contact between unfamiliar conspecifics was prevented by keeping only siblings together in isolated groups. Upon arrival the piglets were grouped—within the same IGEg group—so that each group consisted of six unacquainted piglets in either a barren or an enriched pen. Both housing types measured 6.7 m^2^. The barren pens (**B**) had a 47% slatted floor and a chain with ball hanging from the wall. The enriched pens (**E**) had a solid floor covered with a deep litter layer of straw and wood shavings, and a chain with ball hanging from the wall. Feed was offered *ad libitum* in a feeder with three feeding places. Water was available via a drinking nipple. Per batch, 16 pens of six piglets each were studied, resulting in a total of 80 pens in a 2 × 2 experimental arrangement with IGEg (high vs. low) and housing (barren vs. enriched) as treatments.

### Backtest response pre-weaning

At ~ 14 days of age, piglets were subjected to a backtest following the methods of Hessing et al. (1993) and Bolhuis et al. (2003). In the backtest a piglet is placed in a supine position for one minute, during which the piglet’s response is monitored. The number of struggles, also called escape attempts, together with the number of vocalizations, can give an indication of a piglet’s coping style. Hereby, piglets are commonly classified into either high resisting (HR) or low resisting (LR), based on the distribution of the continuous scale (Iversen et al. 2017). Previously, no relationship between backtest classification and IGEg was found (Reimert et al. 2013). The backtest classification was here predominantly used to standardize group composition, as the behaviour of animals with different coping styles may profoundly differ. Group composition post-weaning was standardized with a LR:HR ratio in accordance with the whole tested population; meaning that each pen contained at least two pigs of each backtest classification and at least one HR male and one HR female (Ursinus et al. 2014a; Camerlink et al. 2013).

### Body weight

Weight gain of piglets in the first 4 days after weaning was used as indicator of an individual’s ability to cope with the stressful conditions surrounding weaning. Body weight of piglets was recorded at weaning (before transport to the experimental facilities; ~ 27 days of age) and 4 days after. Piglets were weighed individually by placing them in a crate onto a weigh scale appropriate for the size of the piglets at that age. Average daily gain (ADG) in g per day (g/d), was calculated by dividing the weight gain by the 4 days.

### Behavioural observations

Behaviour, as an indicator of coping ability, was recorded through video observations. Video cameras were mounted to the ceiling to record the pens in top view with a small angle. Each camera captured two pens at the same time in full colour at 25 frames per sec. Video data were recorded with Geovision during the day of weaning and the day after. For recognition during behavioural observations, piglets received a number sprayed on their back with a stock marker upon entering the pen at weaning. Behaviour was then observed from video by three trained observers. For each pig they recorded the latency (in min.) to explore the feed / eat, to lie down individually and to lie down together with at least two other pigs. See Table 1 for the definitions used for scoring these behaviours. Lying down together with at least two others was chosen so that it would be more than random contact of, for example, the hind legs touching each other. Latencies were recorded from the moment the individual piglet was placed in the pen up to maximum 5 h after. If an animal did not show one of the scored behaviours within 5 h then the latency was set to 5 h (300 min).

**Table 1:** Ethogram for behavioural observations from video

Behaviour	Description	Observation
Exploring/eating feed	Head is in the feed trough ≥ 3 s continuously	By individual
Lying down individually	Belly touches the floor for ≥ 5 s	By individual
Lying down together	Lying in body contact with at least two other pigs	By individual

### Data analysis

Data were analysed with SAS version 9.3. The response variables were weight, ADG, latency to explore feed, latency to lie down, and latency to lie down in a group. From the 480 pigs, 466 were included in the analysis for the latency to explore feed and to lie down individually, whereas the latency to lie down together was recorded for 165 pigs (two batches only). The residuals of weight, ADG, and the latencies to lie down individually and in a group were normally distributed but the residuals of the latency to eat were skewed and had to be log transformed before reaching a normal distribution.

Data were analysed in Generalized Linear Models (PROC MIXED) to include the random effects for batch, group of pigs (pen) and sow (i.e. dam). The fixed effects in each model where the genetic line (i.e., High or Low IGEg) and housing conditions (B / E), their interaction (testing Genotype × Environment, i.e. G × E effects), sex (M / F) and coping style (HR / LR). Values are Least Square Means with SEM unless stated otherwise (e.g. back-transformed means).

## Results

### Weight gain after weaning

**Fig. 1:**
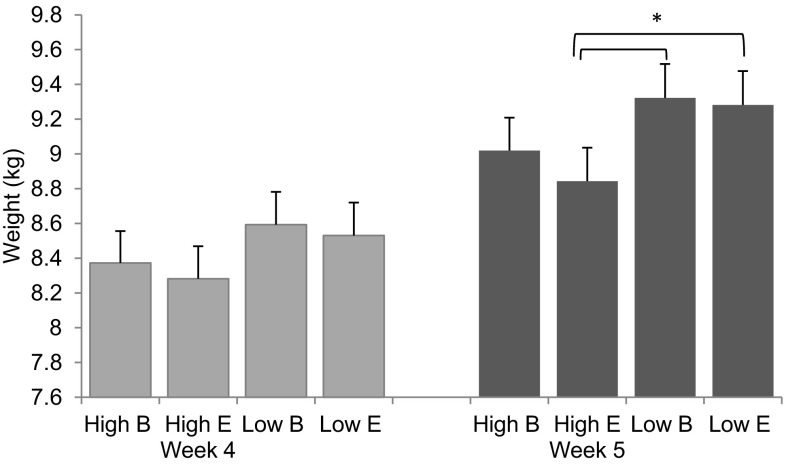
Means with SE for body weight in kg, by IGEg group (High/Low) and housing condition (Barren, B/Enriched, E), at weaning (week 4) and 4 days later (week 5)

At weaning the weight of High and Low IGEg pigs did not differ significantly (*F*_1,335_ = 2.14; *P* = 0.14) (Camerlink et al. 2014). Four days later, piglets of the High IGEg group were lighter than piglets from the Low IGEg group (High 8.93 ± 0.18 kg; Low 9.30 ± 0.19 kg; *F*_1,335_ = 5.08; *P* = 0.02; Fig. 1). The weight difference was also reflected in a lower ADG for High IGEg pigs as compared to Low IGEg pigs in the first 4 days after weaning (High 160 ± 2 g ADG; Low 200 ± 2 g ADG; *F*_1,335_ = 5.69; *P* = 0.02). There was no overall effect of housing conditions, sex or coping style on the weight or ADG at weaning or in the first 4 days after weaning (all *P* > 0.10). There were no significant G × E interactions for weight or ADG.

### Behaviour at weaning

**Fig. 2:**
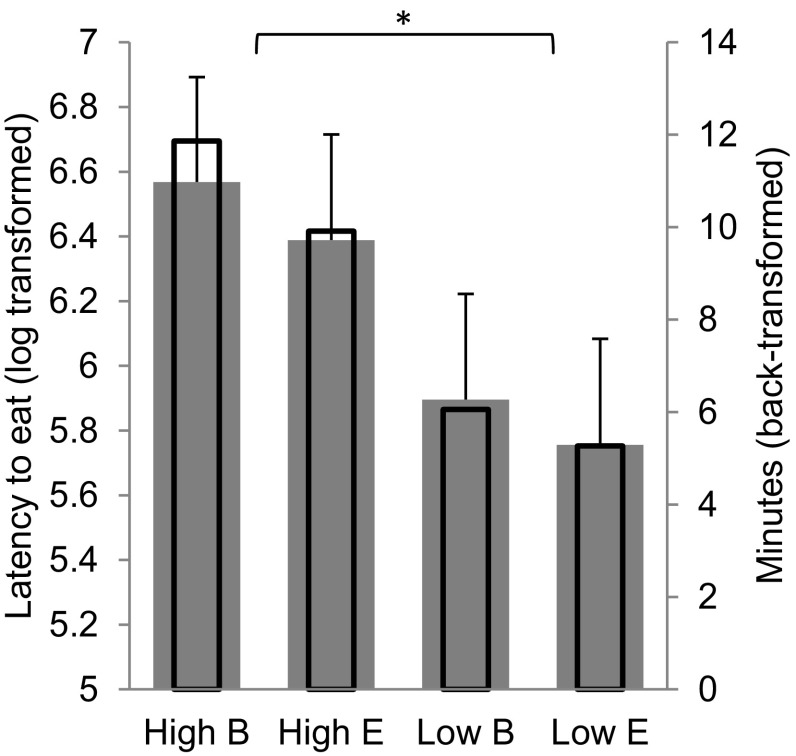
Mean ± SE latency on log transformed scale (grey bars) and back-transformed to minutes (thinner transparent bars) to start exploring or eating the feed after arrival in the experimental facilities (after being weaned and transported), by IGEg group (High / Low) and housing condition (Barren, B / Enriched, E)

On average, pigs started investigating the feed at 17 min (995 ± 65 s; raw means) after arriving into the new facilities (head in the feeder for at least 3 s). This latency to explore or eat the feed was significantly affected by the genetics. High IGEg pigs took double the time compared to Low IGEg pigs before they reached for the feeder High 6.48 ± 0.3 log, 651 s, 11 min; Low 5.83 ± 0.3 log, 339 s, 6 min; *F*_1, 321_ = 17.60; *P* < 0.001; Fig. 2. There were no effects of housing conditions (Fig. 2), sex or coping style, and there was no G × E interaction for the latency to explore feed.

**Fig. 3:**
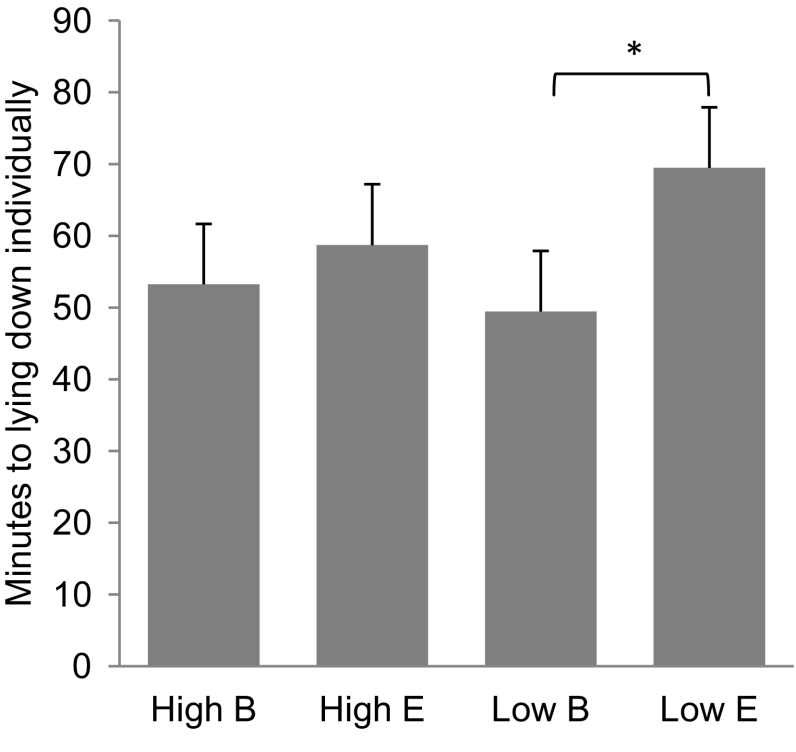
Mean ± SE latency (in minutes) to lie down individually after arrival in the experimental facilities (after being weaned and transported), by IGEg group (High/Low) and housing conditions (Barren, B / Enriched, E)

It took on average 56 ± 2 min before pigs were lying down individually. The genetics did not influence the time to lie down (*F*_1,264_ = 0.48; *P* = 0.49), but pigs in the barren conditions lay down sooner than pigs in straw enriched pens (*F*_1,264_ = 9.79; *P* < 0.01; Fig. 3). Sex and coping style did not influence the latency to lie down individually.

After on average 108 ± 3 min (1¾ h) pigs started to lie down in proximity to at least two others. There was no influence of genetics (*F*_1,105_ = 1.51; *P* = 0.22) or housing conditions (*F*_1,105_ = 1.31; *P* = 0.26) on the latency to lie down in a group. There was no G × E interaction (High B 102 ± 12 min; High E 115 ± 13; Low B 88 ± 11; Low E 101 ± 12; *F*_1,105_ = 0.00; *P* = 0.97), and no influence of sex or coping style.

## Discussion

Pigs divergently selected for Indirect Genetic Effects for growth (IGEg) were housed in either conventional barren pens or straw enriched pens, creating a G × E set-up. Animals were studied around weaning, which is a stressful period for pigs which here coincided with separation from the dam, a 2.5 h journey by truck and a new environment with unfamiliar conspecifics and increased human handling. Low IGEg pigs had a shorter latency to start exploring or eating the feed and a greater weight gain over the first 4 days after weaning. Thus, in contrast with our hypothesis that Low IGEg might be more sensitive to stress, based on behavioural and physiological data at later age, they in fact responded seemingly more relaxed to this stressful situation.

In theory, animals selected for high IGEg during the grow-finisher phase would grow better when kept with group mates with similar social breeding value (as in our experiment), because they receive positive indirect genetic effects from the others in the group. The higher weight gain in Low IGEg, which seems contradictory, is however in line with previous work on IGEg in pigs (Rodenburg et al. 2010; Canario et al. 2012). The aforementioned studies found that pigs with a high breeding value for IGEg showed more aggression when grouped with unfamiliar conspecifics. Aggression at regrouping can result in a depression in growth performance (e.g. Tan et al. 1991), and this would be opposite to the expected positive effect on the growth of group members. At a few weeks after regrouping, however, pigs with a high IGEg showed less aggression than pigs with a low IGEg (Rodenburg et al. 2010; Canario et al. 2012). The former studies suggest that selection on high IGEg contributes to a better establishment of a stable dominance hierarchy, and thereby result in less aggression over the long term.

Previously reported data on the current study population showed that on the day after weaning the genetics did not influence the aggressive behaviour, but at 3 days after weaning High IGEg pigs indeed showed less aggression than Low IGEg (Camerlink et al. 2013). In addition, at later age High IGEg pigs showed less aggression upon reunion with group mates after 24 h separation for a regrouping test (Camerlink et al. 2013). Thus, the smaller weight gain of High IGEg pigs at weaning as well as the longer latency to explore or eat the feed may be related to High IGEg pigs investing more time into establishing social relationships upon first encounter.

Physiological factors that may differ due to selection on IGEg may, however, co-exits alongside any potential differences in the formation of dominance relationships. Based on additional behavioural and physiological data from the same animals in later life we here discuss the potential of alternative explanations.

Current commercial pig breeds have mostly been selected on fast growth and a high feed intake (Rauw et al. 1998). Highly productive animals will have a high, potentially unfulfilled, metabolic demand causing a strong motivation to eat (D’Eath et al. 2009). If this food motivation cannot be fulfilled then this may result in amongst others redirected oral manipulative behaviour and stereotypies (D’Eath et al. 2009; Ursinus et al. 2014c). When this oral manipulative biting is (re)directed towards conspecifics, the social interactions will become harmful, which may be reflected in IGEg (Turner 2011). Low IGEg pigs indeed showed higher levels of oral manipulative behaviours towards conspecifics and objects in the environment later in life (Camerlink et al. 2015). This may suggest that Low IGEg piglets might have a higher internally driven food motivation which would explain the short latency to start exploring or eating the feed despite the new environment and other likely stressful factors around weaning.

The above mentioned traits—oral manipulative behaviour, food motivation and growth—may be associated with the amino acid tryptophan. Tryptophan is an essential amino acid which plays a role in amongst others appetite, body protein deposition, growth performance and behaviour (Le Floc’h et al. 2011; Sève et al, 1991; Martınez-Trejo et al. 2009; Azmitia 2010). Tryptophan is a precursor for serotonin, and physiological data from the same animals as studied here showed variance in the functioning of the serotonergic system. Low IGEg pigs tended to have a higher serotonin platelet uptake velocity at 22 weeks of age compared to High IGEg pigs (untransformed LSmeans High IGEg = 41.6; Low IGEg = 43.6 pmol/10^9^ platelets/min, SE = 1.4; *F*_1,332_ = 3,09; *P* = 0.08; unpublished results from the study described in Ursinus et al. 2014b). A higher platelet uptake velocity may imply that free serotonin in blood plasma is only shortly available to use and is quickly stored in blood platelets. The serotonergic system, which relates to the amount of available tryptophan, is known to be involved in stress-related disorders (e.g. Delorme et al. 2005; Coppen et al. 1976; Cleare 1997) and has found to be associated with oral manipulative behaviour in pigs (tail biting) (Ursinus et al. 2014b; Valros et al. 2015). Our findings suggest that high IGEg pigs may have a higher availability or lower utilization of the serotonin precursor tryptophan, which could explain the lower occurrence of oral manipulative behaviours.

The enteric microbiota has been suggested to play a role, through the gut-brain axis, in the intensity of oral manipulative behaviours in livestock (Brunberg et al. 2016). The host’s genetics influence the composition and functioning of the gut microbiota (Hansen et al. 2010; pigs:; Zimmermann et al. 2016) and it is therefore worth considering the potential influence of genetic selection for IGEg on gut microbiota, as an alteration would affect both behaviour and growth (Cryan and Dinan 2012; Pascoe et al. 2017).

This study kept animals in two contrasting housing conditions to explore potential genotype-by-environment (G × E) interactions. Half of the pigs were kept on a deep layer of straw, which is for pigs extremely appealing to explore and chew on (Fraser et al. 1991; Jensen et al. 2015) and may in some cases increase growth performance, although results are inconsistent about the effects on growth (reviewed by Millet et al. 2005). It thus created an additional novelty to explore, which may have made the animals more directed toward the environment rather than to explore the feed or to interact with conspecifics. In enriched pens, pigs were indeed more exploring, rooting, playing and chewing on straw in the first week after weaning (Camerlink et al. 2015). Hence, this may explain the longer latency to lie down in straw enriched pens, although it did not increase the latency to start eating. There were no G × E interactions for the behaviour and productivity at this age.

IGEg are currently being explored or taken into account by most of the major pig breeding companies. One aim of including IGE into breeding estimates is to gain a more accurate prediction of breeding values, which allows for an increase in response to selection. Theory predicts that a group of animals selected for high IGEg will grow better due to the positive effect on each other’s growth (Bijma et al. 2007). In our experiment, parents of the pigs had been selected for their estimated IGE on growth during the entire fattening period. Our results suggest that the outcome of such selection may result in temporarily reduced growth, which could be due to aggression during the formation of dominance relationships. Stable dominance relationships can be beneficial in the long run and reduce the amount of aggression and unrest later in life (Turner et al. 2017). The effect of selection for IGEg on behaviour should therefore actually not be assessed based on a single time frame (Schneider et al. 2017). Rather, social interactions between group living species are dynamic rather than static, and the current explorations on combing the fields of social network analysis with IGE modelling are a promising avenue for further research.

## Conclusion

Pigs divergently selected on Indirect Genetic Effects for growth (IGEg) showed differences in their behaviour and weight gain in the first 4 days after weaning. High IGEg animals were slower to explore the new feed and grew slower in the first 4 days after weaning than Low IGEg pigs. Although this seems opposite to the theory of selection for IGEg, the results are in line with previous studies which suggested that in newly formed groups high IGEg pigs may invest more energy in social interactions including aggression.
